# CD64 and Group II Secretory Phospholipase A2 (sPLA2-IIA) as Biomarkers for Distinguishing Adult Sepsis and Bacterial Infections in the Emergency Department

**DOI:** 10.1371/journal.pone.0152065

**Published:** 2016-03-22

**Authors:** Toh Leong Tan, Nurul Saadah Ahmad, Dian Nasriana Nasuruddin, Azlin Ithnin, Khaizurin Tajul Arifin, Ida Zarina Zaini, Wan Zurinah Wan Ngah

**Affiliations:** 1 Department of Emergency Medicine, Faculty of Medicine, Universiti Kebangsaan Malaysia, Kuala Lumpur, Malaysia; 2 Department of Pathology, Faculty of Medicine, Universiti Kebangsaan Malaysia, Kuala Lumpur, Malaysia; 3 Department of Biochemistry, Faculty of Medicine, Universiti Kebangsaan Malaysia, Kuala Lumpur, Malaysia; Rutgers University, UNITED STATES

## Abstract

**Introduction:**

Early diagnosis of sepsis and bacterial infection is imperative as treatment relies on early antibiotic administration. There is a need to develop new biomarkers to detect patients with sepsis and bacterial infection as early as possible, thereby enabling prompt antibiotic treatment and improving the survival rate.

**Methods:**

Fifty-one adult patients with suspected bacterial sepsis on admission to the Emergency Department (ED) of a teaching hospital were included into the study. All relevant cultures and serology tests were performed. Serum levels for Group II Secretory Phospholipase A2 (sPLA2-IIA) and CD64 were subsequently analyzed.

**Results and Discussion:**

Sepsis was confirmed in 42 patients from a total of 51 recruited subjects. Twenty-one patients had culture-confirmed bacterial infections. Both biomarkers were shown to be good in distinguishing sepsis from non-sepsis groups. CD64 and sPLA2-IIA also demonstrated a strong correlation with early sepsis diagnosis in adults. The area under the curve (AUC) of both Receiver Operating Characteristic curves showed that sPLA2-IIA was better than CD64 (AUC = 0.93, 95% confidence interval (CI) = 0.83–0.97 and AUC = 0.88, 95% CI = 0.82–0.99, respectively). The optimum cutoff value was 2.13μg/l for sPLA2-IIA (sensitivity = 91%, specificity = 78%) and 45 antigen bound cell (abc) for CD64 (sensitivity = 81%, specificity = 89%). In diagnosing bacterial infections, sPLA2-IIA showed superiority over CD64 (AUC = 0.97, 95% CI = 0.85–0.96, and AUC = 0.95, 95% CI = 0.93–1.00, respectively). The optimum cutoff value for bacterial infection was 5.63μg/l for sPLA2-IIA (sensitivity = 94%, specificity = 94%) and 46abc for CD64 (sensitivity = 94%, specificity = 83%).

**Conclusions:**

sPLA2-IIA showed superior performance in sepsis and bacterial infection diagnosis compared to CD64. sPLA2-IIA appears to be an excellent biomarker for sepsis screening and for diagnosing bacterial infections, whereas CD64 could be used for screening bacterial infections. Both biomarkers either alone or in combination with other markers may assist in decision making for early antimicrobial administration. We recommend incorporating sPLA2-IIA and CD64 into the diagnostic algorithm of sepsis in ED.

## Introduction

Sepsis is a condition in which patients develop systemic inflammatory response syndrome (SIRS) associated with infection [1]. Sepsis results in 14000 estimated cases annually in the Emergency Department (ED) of Universiti Kebangsaan Malaysia Medical Centre (UKMMC), a tertiary teaching hospital. Our hospital’s prevalence of sepsis is 25–35% based on our yearly census from the year 2013 to 2014. The annual mortality of sepsis is 13–16%. The diagnosis of sepsis is a challenge, as there is no single reliable test for its early confirmation or exclusion. The ability to perform risk stratification early in the patient’s course of illness may guide physicians to a more effective management, improve patient outcome and reduce the mortality and morbidity of sepsis [2].

Blood culture has been the gold standard to detect bacterial infections. However, it has a low sensitivity and using it to diagnose bacteraemia has its own set of challenges [3,4]. Furthermore, this procedure requires 48 hours before results are available to indicate bacteraemia. Other biomarkers that may assist in the diagnosis of sepsis includes serum procalcitonin (PCT) and C-reactive protein (CRP). PCT has been proposed to be a more specific [5] and better prognostic [6] marker than CRP. However, both biomarkers have been shown to possess low specificity and sensitivity [7,8], making the diagnosis of sepsis challenging. Therefore, a continuous search for other candidate biomarkers for sepsis is needed. A recent systematic review analyzed 178 different biomarkers from 3370 studies involved in sepsis. Out of the 178 biomarkers, five of these reported sensitivity and specificity of more than 90%; they are IL-12, Interferon-induced protein 10(IP-10), Group II phospholipase A2 (sPLA2-IIA), neutrophil CD11b, and CD64 [9]. Among these biomarkers, CD64 and sPLA2-IIA were suggested to be the best to indicate bacteraemia in sepsis.

CD64 (FcgRI), is one of the Fc receptors for IgG constitutively present on macrophages, monocytes, eosinophils, and neutrophils. During an infection, studies have shown that there is an increased in the CD64 expression in the presence of microbial wall components, complement split products, and some pro-inflammatory cytokines, such as granulocyte colony-stimulating factor (G-CSF) and interferon gamma (IFN-Ƴ) [10–12]. On the other hand, the expression is significantly decreased when these stimulation factors were removed, resulting in the decline of CD64 activity within 48 hours and a return to normal baseline levels after 7 days [13].

**Fig 1:**
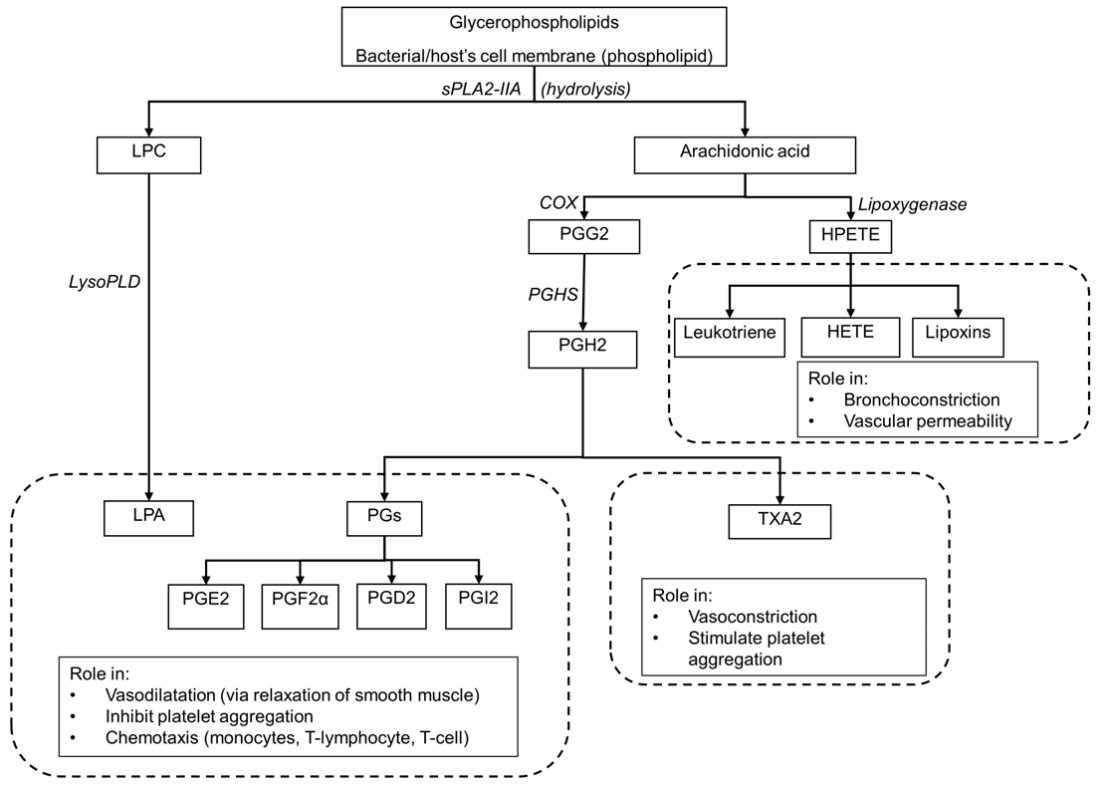
Schematic diagram outlining the fate of glycerophospholipid following hydrolysis by sPLA2-IIA. Hydrolysis by sPLA2-IIA results in production of LPC and AA, which leads to generation of various pro-inflammatory metabolites. Abbrev: LPC, lysophosphotidylcholine; LysoPLD, lysophospholipase D; LPA, lysophosphatidic acid; COX, cyclooxygenase; PGG2, prostaglandin G2; PGHS, prostaglandin H synthase; PGH2, Prostaglandin H2; PGs, prostaglandins; PGE2, prostaglandin E2, PGF2α, prostaglandin F2α; PGD2, prostaglandin D2; PGI2, prostaglandin I2 also known as prostacyclin; HPETE, 5-hydroperoxyeicosatetraenoic acid; HETE, Hydroxyicosatetraenoic acid; TXAs, thromboxanes.

Apart from tissue injury, cell damage and irritant exposure, infection can also trigger the inflammation pathway. One of the immediate responses to inflammation is the hydrolysis of the phospholipid group on the membrane lipids by the enzyme phospholipase A2 (PLA2), which belongs to a family of acute phase proteins [14]. Phospholipids such as glycerophospholipid on a cell membrane consists of 3-carbon chains. Hydrolytic activity of PLA2 (Fig 1) results in the removal of the fatty acid on carbon 2, which is frequently arachidonic acid [15,16]; leaving behind lysophosphatidylcholine (LPC) [17,18]. Lysis of LPC in turn will start a cascade of reaction which ultimately leads to not only the inflammation process, but also direct bactericidal activity in sepsis [17,18]. The metabolism of arachidonic acid leads to the generation of arachidonic acid metabolites, catalyzed by various enzymes. All of these metabolites are pro-inflammatory mediators; they include 5-hydroperoxyeicosatetraenoic acid (HPETE, generated by activity of lipoxygenase) [19,20] and prostaglandin G2 [21,22] (catalyzed by cyclooxygenase). HPETE can be converted further into leukotrienes [23] and hydroxyicosatetraenoic acid (HETE) [24], while prostaglandin G2 can be catalyzed further to generate thromboxanes (thromboxane A2 and B2) [25], prostacyclin (PGI2) [26] and various forms of prostaglandins (prostaglandins D2, E2 and F2α) [27] which will further amplify the inflammation signals [28–31].

PLA2 has a number of subfamilies of enzymes, one of which includes secreted PLA2 (sPLA2). sPLA2 exist as ten active isoforms, differing in the source of organisms and sites of activity [32–36]. One isoform; sPLA2-IIA, has been identified as exhibiting sensitivity and specificity of more than 90% towards sepsis [9]. Coined as a bactericidal enzyme, the catalytic activity of sPLA2-IIA is thought to be its prominent role via hydrolysis of bacterial membranes [37–39]. Interestingly, even when the active site has been mutated, sPLA2-IIA still exhibited anti-bacterial property [40].

Similar with CD64, sPLA2-IIA expression in humans is also increased during infection [38]. The inflammatory response results in numerous physiological responses such as vascular dilatation [41], inhibition of platelet aggregation [15] and chemotaxis [42, 43] (Fig 1). Earlier studies have suggested that the levels of sPLA2-IIA correlated well with the severity of septic shock and its outcome. It also reflected the severity of inflammation in infections and non-infectious inflammatory conditions [44–46]. The levels of sPLA2-IIA appeared useful in measuring the degree of inflammation in various bacteraemic and non-bacteraemic infections [47–50] and it might also help in distinguishing between bacterial and viral infections [47]. The levels of sPLA2-IIA have been found to be higher in patients with septic shock than in those without [51]. High or persistently elevated levels of sPLA2-IIA have also been shown to be associated with adverse outcomes in sepsis [48,49]. Intriguingly, a study investigating the effect of anti-sPLA2-IIA compared to placebo in reducing 28-day mortality in severely septic patients did not show overall survival benefit [52].

The aim of the present study was to evaluate the performance of CD64 and sPLA2-IIA as biomarkers in the diagnosis of sepsis, and whether these markers can be used to differentiate between bacterial and non-bacterial infection.

## Methods

### Patient Recruitment

The study was conducted over a period of 10 months (March to December 2014), after obtaining approval from Universiti Kebangsaan Malaysia Research Ethics Committee (Ethic code: FF-2014-150). Written consent was obtained from all subjects. No minor was recruited into the study; all subjects were 18 years old and above. This single-centered prospective observational study consisted of consented patients who presented to the ED of UKMMC, which is a 1000-bed urban academic hospital with 72000 ED visits annually. All patients with suspected sepsis and also those who had a minimum of two SIRS criteria, were consecutively included into this study [1]. We also included patients with systolic blood pressure (SBP) less than 90mmHg after a minimum of 30ml/kg crystalloid fluid bolus. Exclusion criteria included patients who have been partially treated with antibiotics for more than 3 days, patients with ongoing oncology diseases, patients who passed away during the period of recruitment, and patients who were transferred to other hospitals. Blood samples were collected for CD64 & sPLA2-IIA measurements. Relevant cultures and serology tests for all patients were carried out. Bacterial infection was defined as clinical bacterial infection or positive bacterial culture, while non- bacterial infection was defined as clinical infection with negative bacterial culture or positive serology test for non-bacterial pathogen. Sepsis was defined as SIRS with clinical suspicion of infection and positive culture or serology test result.

### Determination of CD64 expression

Measurement of neutrophil CD64 expression was done via staining of 50μl whole blood with a combination of both anti-CD64-PE and anti-CD45-PerCP (Becton-Dickinson, San Jose, CA). The sample was then left for 60 minutes in the dark and an additional 60 minute incubation to reduce non-specific background staining. All samples were analyzed on a FACScan flow cytometer (Becton-Dickinson), where a threshold of FL-3 was used to identify leucocytes. The results were expressed as antibodies bound per cell (abc).

### Determination of sPLA2-IIA levels

sPLA2-IIA activity in serum was detected by using the sPLA2-IIA (human type IIA) Enzyme Immunometric Assay Kit (Cayman Chemical, USA) according to manufacturer’s instructions. sPLA2-IIA levels in serum samples were tested in triplicates and determined against the standard curve of each EIA assay. All wells were read at a wavelength between 405 and 420nm.

### Statistical analyses

Statistical analyses were executed using SPSS software^TM^. Median was determined from the interquartile range. Mann-Whitney *U* test was used to test for differences in analyzed parameters between groups. An α value of less than 0.05 for a two-tailed test was considered significant. We plotted receiver operating characteristics (ROC) curves and evaluated the area under the curve (AUC) of each selected variable to measure the power of each assay in discriminating between sepsis and non-sepsis groups, as well as bacterial and non-bacterial infection. The cutoff points of each variable were then determined. Accuracy for the parameters was determined using Cross table for the Accuracy and Kappa agreement test.

## Results

From March to Dec 2014, we screened a total of 1320 patients who presented to the ED with SIRS. With the study’s strict recruitment criteria, only a total of 69 patients were eligible for the study, of which 51 patients were selected for analysis after exclusion. A total of 18 patients were excluded for various reasons (eight of them have been partially treated with antibiotic; four of them had malignancy; four of them had concurrent viral and bacterial co-infection; one patient had end-stage renal failure and one had ongoing myocardiac infarction). Among these recruited patients, 42 of them presented with sepsis while 21 of them had culture-confirmed bacterial infections. Demographic data of the recruited patients is shown in Table 1. Bacterial aetiology as detected by cultivation is shown in Table 2.

**Table 1:** Demographic data of recruited patients.

	Total patients (n = 51)
Age (years; mean ± SD)	53.7 ± 20.8
Gender	
Male	26(54%)
Female	25(46%)
Clinical Characteristic	
Systolic Blood pressure	130 ± 34
Diastolic Blood Pressure	75 ± 21
Heart Rate (per minute)	110 ± 22
Respiratory Rate (per minute)	26 ± 9
Temperature (° Celsius)	38.3 ± 1.0
Total White Cell Count (x 10^9^)	12.0 ± 8.0
Sepsis	42 (82.4%)
Non-sepsis	9 (17.6%)
Source of infection	
Respiratory	12 (23.5%)
Musculoskeletal	2 (3.9%)
Urinary	5 (9.8%)
Dengue infection	6 (11.8%)
Gastrointestinal	8 (15.7%)
Blood/Catheter related	3 (5.9%)
Central Nervous	1 (2.0%)
Bacterial Blood Culture, Positive	13(25.5%)
Bacterial Culture, Positive	8 (15.7%)
Dengue Serology, Positive	6 (11.8%)

**Table 2:** Bacterial aetiology as detected via Cultivation.

Cultured Organisms	Frequency
*Escherichia coli*	5
*Escherichia coli* ESBL^a^	2
*Staphylococcus pyogenes*	2
*Aeromonas hydrophila*	1
*Bacteroides fragilis*	1
*Enterobacter cloacae*	1
*Enterobacter species*	1
*Klebsiella pneumoniae*	1
*Klebsiella species ESBL*	1
Methicillin-Resistant *Staphylococcus aureus*	1
*Proteus species*	1
*Pseudomonas aeruginosa*	1
*Staphylococcus aureus*	1
*Streptococcus* Group B	1
*Streptococcus viridans*	1

^a^ Extended-spectrum beta-lactamases

**Fig 2:**
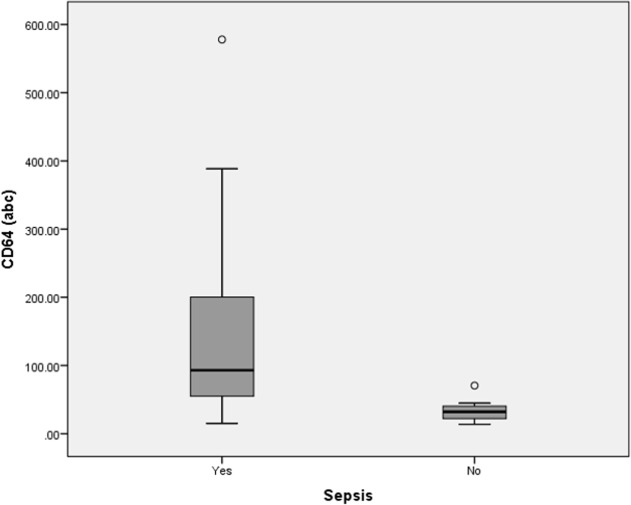
Box-plot for CD64 levels in sepsis and non-sepsis diagnosis. abc = antigen bound cell. Boxes show the 25^th^-75^th^ centiles, while whiskers indicate the 10^th^ and 90^th^ centiles. Horizontal lines within the boxes indicate the median. Outliers are shown as circles.

**Fig 3:**
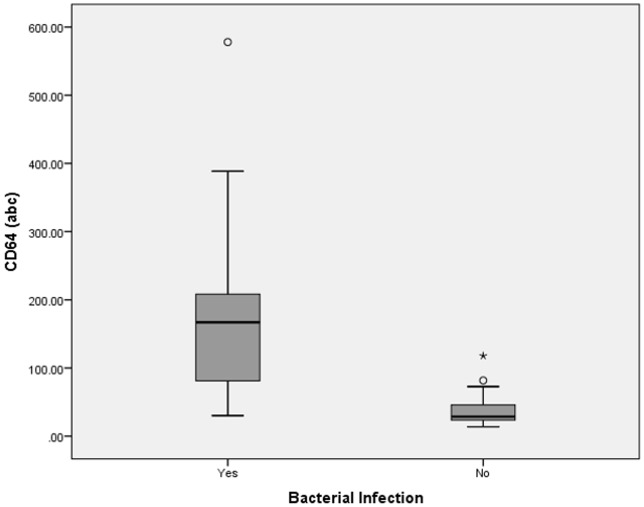
Box-plot for CD64 levels in bacterial and non-bacterial infection diagnosis. abc = antigen bound cell. Boxes show the 25^th^-75^th^ centiles, while whiskers indicate the 10^th^ and 90^th^ centiles. Horizontal lines within the boxes indicate the median. Outliers are shown as circles and stars.

CD64 levels of both sepsis and bacterial infection groups had non-parametric distributions. Median for CD64 levels (93 ± 122abc) were significantly higher in the sepsis group compared to the non-sepsis group (p = 0.001, Mann-Whitney *U* test) (Fig 2). With the cutoff point of 45 abc, CD64 was able to distinguish between sepsis from non-sepsis group. It had a specificity of 89% and sensitivity of 81%. The positive predictive value was 97% and the negative predictive value was 50%, making it a very good biomarker for sepsis (ROC, AUC = 0.88, 95% confidence interval (CI) = 0.82–0.99, Accuracy = 0.82, Kappa = 0.54) (Table 3). CD64 levels (median = 167 ± 121abc) for both bacterial and non-bacterial infection groups showed statistical significance (p = 0.001, Mann-Whitney *U* test) (Fig 3). We suggest that at the cutoff point of 46abc, CD64 was able to diagnose bacterial infection. Sensitivity and specificity were 94% and 83%, respectively; while the positive and negative predictive values were 91% and 88%, respectively. CD64 was found to have excellent accuracy in diagnosing bacterial infection (ROC, AUC = 0.95, 95%CI = 0.93–1.00, Accuracy = 0.90, Kappa = 0.78).

**Table 3:** CD64 and sPLA2-IIA expression for recruited patients. sPLA2-IIA = Group IIA secretory phospholipase A2; abc = antigen bound cell; AUC = area under the curve; Sn = Sensitivity; Sp = Specificity; PPV = positive predictive value; NPV = negative predictive value; CI = Confidence Interval.

8Biomarkers	AUC (CI = 95%)	Cut-off point	Sn (%) (CI = 95%)	Sp (%) (CI = 95%)	PPV (%) (CI = 95%)	NPV (%) (CI = 95%)	Accuracy	Kappa, κ
**Sepsis versus non-sepsis patients**								
CD64 (abc)	0.88 (0.82–0.99)	45	81 (66–91)	89 (52–100)	97 (85–100)	50 (25–75)	82	0.54
sPLA2-IIA (μg/l)	0.93 (0.83–0.97)	2.13	91 (77–97)	78 (40–98)	95 (83–99)	64 (31–89)	88	0.63
**Bacterial infection versus non-bacterial infection patients**								
CD64 (abc)	0.95 (0.93–1.00)	46	94 (80–99)	83 (59–96)	91 (76–98)	88 (64–99)	90	0.78
sPLA2-IIA (μg/l)	0.97 (0.85–0.96)	5.63	94 (79–99)	94 (72–100)	97 (84–100)	90 (67–99)	94	0.87

**Fig 4:**
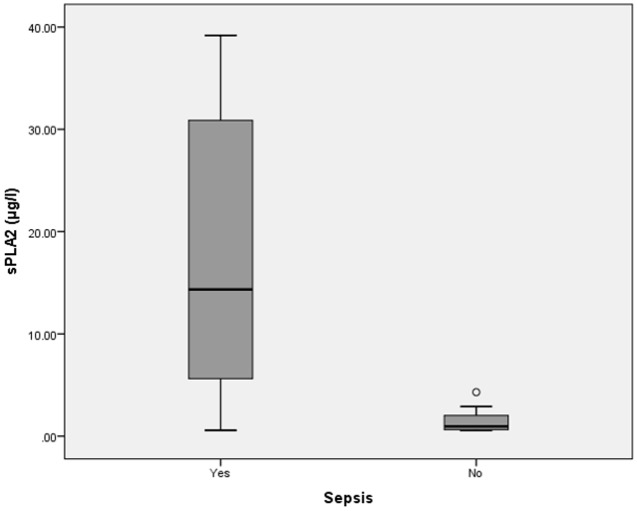
Box-plot for sPLA2-IIA levels in sepsis and non-sepsis diagnosis. Boxes show the 25^th^-75^th^ centiles, while whiskers indicate the 10th and 90th centiles. Horizontal lines within the boxes indicate the median. Outliers are shown as circles.

**Fig 5:**
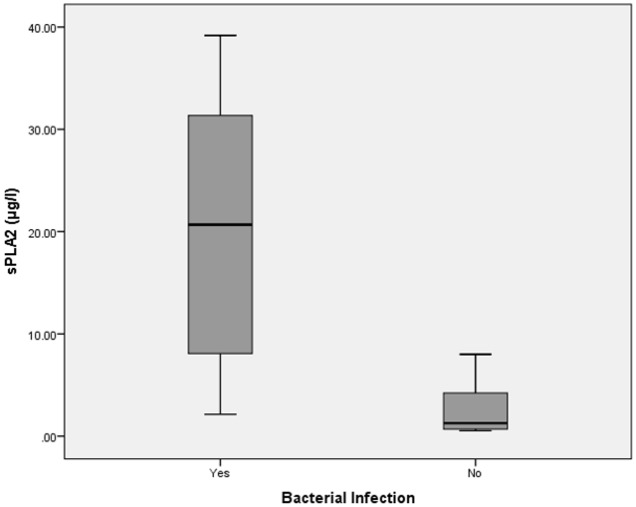
Box-plot for sPLA2-IIA levels in bacterial and non-bacterial infection diagnosis. Boxes show the 25^th^-75^th^ centiles, while whiskers indicate the 10^th^ and 90^th^ centiles. Horizontal lines within the boxes indicate the median. Outliers are shown as circles.

**Fig 6:**
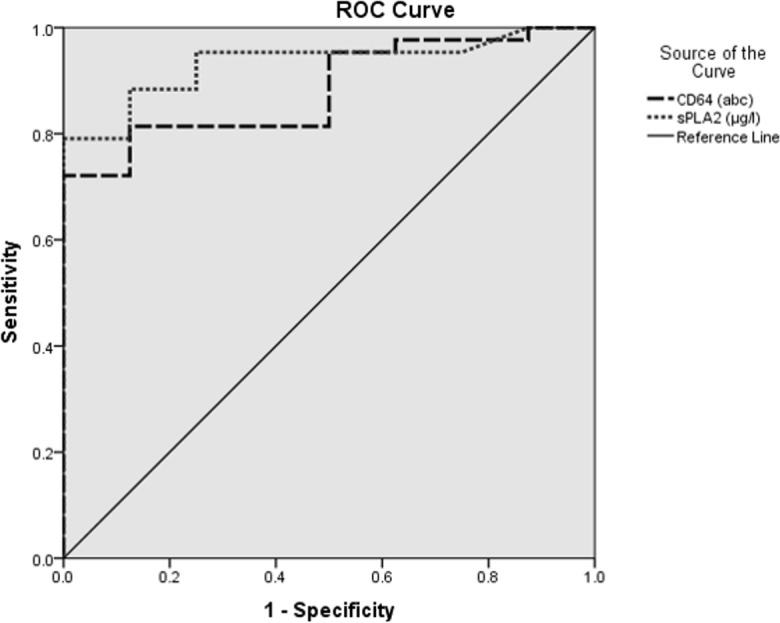
ROC curves for CD64 and sPLA2-IIA in sepsis diagnosis. abc = antigen bound cell.

**Fig 7:**
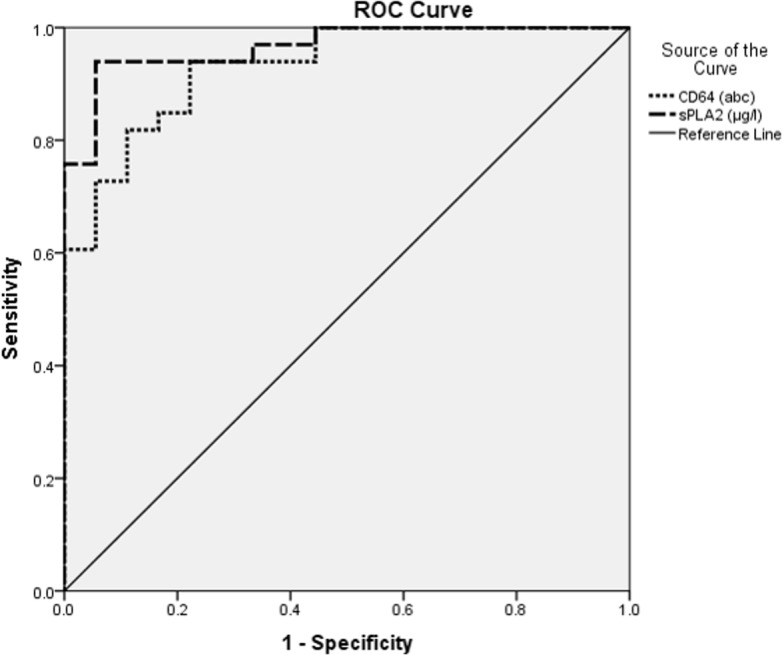
ROC curves for CD64 and sPLA2-IIA in bacterial infection diagnosis. abc = antigen bound cell.

Interestingly, sPLA2-IIA levels also demonstrated a strong correlation with early sepsis diagnosis in adults (median 14.5 ± 12.8μg/l, p = 0.001, Mann-Whitney *U* test) (Fig 4). A cut off level of 2.13μg/l was able to distinguish the sepsis group from non-sepsis group (sensitivity = 91%; specificity = 78%; positive predictive value = 95%; negative predictive value = 64%). sPLA2-IIA was able to accurately diagnose sepsis in adults (ROC, AUC = 0.93, 95%CI = 0.83–0.97, Accuracy = 0.88, Kappa = 0.63) (Table 3). From this interim analysis, we discovered that sPLA2-IIA showed a positive statistical correlation in diagnosing bacterial infection (median = 20.67 ± 11.79 μg/l, p = 0.001, Mann-Whitney *U* test) (Fig 5). The cutoff level of sPLA2-IIA was higher in diagnosing bacterial infection compared to sepsis. This makes sPLA2-IIA a highly accurate biomarker for both screening and diagnosing bacterial infection (ROC, AUC = 0.97, 95% CI = 0.85–0.96, Accuracy = 0.94, Kappa = 0.87). Sensitivity and specificity were 94% and 94%, respectively; while the positive predictive value and negative predictive values were 97% and 90%, respectively. Figs 6 and 7 show ROC curves for both CD64 and sPLA2-IIA levels according to sepsis and bacterial infection diagnoses.

## Discussion

In this study, CD64 showed high specificity and positive predictive value in distinguishing sepsis from non-sepsis groups, making it an accurate biomarker for this purpose. A recent meta-analysis was done in year 2010 to evaluate the diagnostic precision of neutrophil CD64 expression in identifying bacterial infection, which showed a pooled sensitivity of 0.79 and pooled specificity of 0.91 [13]. Shan li et al. (2013) repeated similar neutrophil CD64 expression meta-analysis with a larger sample size estimate pooled 0.76 (95% CI 0.74–0.78) for sensitivity and 0.85 (95% CI 0.83–0.86) for specificity [53]. However, as both meta-analysis involved various methods, this may have contributed to the poor sensitivity of their analysis. In contrast, our study showed that CD64 demonstrated a higher sensitivity as compared to specificity in screening and diagnosing bacterial infection; this is most probably due to the strict adherence of our patient recruitment protocol. We found neutrophil CD64 expression to have a good overall diagnostic performance, and that this biomarker could be a promising and evocative biomarker to screen for bacterial infection in ED [53–57].

Results from our study also demonstrated sPLA2-IIA as an excellent screening biomarker for sepsis, with high sensitivity and specificity to diagnose bacterial infection. This biomarker is highly accurate and it might be a promising tool to be used in ED to facilitate early diagnosis of sepsis and bacterial infection. This present study is in agreement with Rintala et al. [58] that sPLA2-IIA expression correlated well with sepsis severity and was able to indicate bacterial infection. The lower median of sPLA2-IIA found in our study may be due to our larger sample size compared to Rintala’s study. In 2001, Rintala et al.[59] repeated the study done in 1993 in patients who presented to the hospital in less than 24 hours, and demonstrated that sPLA2-IIA had similar sensitivity and specificity of 80% and an AUC of 0.84. Comparatively, we found that sPLA2-IIA had much better and a higher sensitivity of 94% and specificity of 94% within the 1st hour of ED visit for sepsis and bacterial infection diagnosis.

In comparison with CD64, sPLA2-IIA showed better performance and higher accuracy in diagnosing both sepsis and bacterial infection. In the acute phase of the host inflammatory response during sepsis, sPLA2 enzymes, including sPLA2-IIA is mostly associated with high-density lipoproteins (HDL) [60], which are the major source of phospholipids in plasma. Interestingly, these sPLA2- modified HDL shows potent anti-inflammatory activities [61,62], where they activate neutrophils and trigger the whole anti-inflammatory cascade. We suspect sPLA2-IIa is the initiator molecule in this cascade and plays a crucial part in the host response towards containing sepsis; therefore, whilst the sPLA2-IIA could be used as a potent biomarker for sepsis diagnosis, anti-sPLA2-IIA was not found to provide survival benefit in septic patients [52].

## Conclusion

Taking it all together, sPLA2-IIA showed superior overall performance compared to CD64 in diagnosing sepsis and bacterial infection, and could be used as a good biomarker for this purpose, either singly or in combination with other biomarkers. It may assist clinicians in their decision making for early antimicrobial administration, enable risk stratification and expedite the execution of sepsis bundle. We recommend that future studies with larger sample size for these two promising biomarkers be carried out to validate their diagnostic performance, and to determine if they should be included in the diagnostic algorithm of sepsis management in ED.
